# Prophylactic Use of Macrolide Antibiotics for the Prevention of Chronic Obstructive Pulmonary Disease Exacerbation: A Meta-Analysis

**DOI:** 10.1371/journal.pone.0121257

**Published:** 2015-03-26

**Authors:** Wentao Ni, Xiaodi Shao, Xuejiu Cai, Chuanqi Wei, Junchang Cui, Rui Wang, Youning Liu

**Affiliations:** 1 Department of Respiratory Diseases, Chinese People’s Liberation Army General Hospital, Beijing, China; 2 Department of Clinical Pharmacology, Chinese People’s Liberation Army General Hospital, Beijing, China; University of Dundee, UNITED KINGDOM

## Abstract

**Background:**

Acute exacerbations of chronic obstructive pulmonary disease (AECOPDs) can lead to high frequencies and rates of hospitalization and mortality. Macrolides are a class of antibiotics that possess both antimicrobial and anti-inflammatory properties. Since the occurrence of AECOPDs is associated with aggravation of airway inflammation and bacterial infections, prophylactic macrolide treatment may be an effective approach towards the prevention of AECOPDs.

**Methods:**

We systemically searched the PubMed, Embase and Cochrane Library databases to identify randomized controlled trials (RCTs) that evaluated the effect of prophylactic macrolide therapy on the prevention of AECOPDs. The primary outcomes were the total number of patients with one or more exacerbations as well as the rate of exacerbations per patient per year.

**Results:**

Nine RCTs comprising 1666 patients met the inclusion criteria. Pooled evidence showed macrolides could reduce the frequency of exacerbations in patients with COPD by both unweighted (RR = 0.70; 95% CI: 0.56–0.87; P < 0.01) and weighted approaches (RR = 0.58, 95% CI: 0.43–0.78, P < 0.01). Subgroup analysis showed only 6–12 months of erythromycin or azithromycin therapy could be effective. Moreover, among studies with 6–12 months of azithromycin therapy, both the daily dosing regimen and the intermittent regimen significantly reduced exacerbation rates. The overall number of hospitalizations and the all-cause rate of death were not significantly different between the treatment and control groups. A tendency for more adverse events was found in the treatment groups (OR = 1.55, 95%CI: 1.003–2.39, P = 0.049).

**Conclusions:**

Our results suggest 6-12 months erythromycin or azithromycin therapy could effectively reduce the frequency of exacerbations in patients with COPD. However, Long-term treatment may bring increased adverse events and the emergence of macrolide-resistance. A recommendation for the prophylactic use of macrolide therapy should weigh both the advantages and disadvantages.

## Introduction

About 5%-10% of the adult world population is suffering from chronic obstructive pulmonary disease (COPD) [1]. It is the fourth leading cause of death and nearly 2.79 million patients die of this debilitating disease per year in middle-income countries [2]. COPD is characterized by the episodes of exacerbations described as an acute worsening of respiratory symptoms. The exacerbations consist of worsening of symptoms such as frequency and severity of cough and increases or change in the volume or character of sputum [3]. Acute exacerbations of chronic obstructive pulmonary disease (AECOPDs) can significantly influence the natural course of the disease, leading to a decrease in the health-related quality of life, frequent hospitalizations and higher mortality rates [4]. In addition, AECOPDs are estimated to account for as much as 50–75% of the total costs for COPD treatment [5]. Therefore, it is of great significance to take effective measures to prevent COPD exacerbations.

At present, there are mainly two strategies used for the prevention of AECOPDs. One is non-pharmacologic interventions including smoking cessation, influenza vaccination, pulmonary rehabilitation and lung volume reduction surgery [6]. The other is pharmacologic interventions such as long-acting bronchodilators alone or combined with inhaled corticosteroids, phosphodiesterase-4 inhibitors and N-acetylcysteine [6]. However, these strategies reduce AECOPDs at best by 25–30% and approximately one-third COPD patients still experience one or more exacerbations every year [7].

Considering that more than 50% of exacerbations are due to airway bacterial infections, and the exacerbations are almost always accompanied by an increase in airway inflammation [8, 9], prophylactic usage of macrolides that possess both anti-bacterial and anti-inflammatory effects may be an effective approach to prevent AECOPDs. To confirm this hypothesis, several randomized controlled trials (RCTs) were launched, yielding various results. Prior meta-analyses evaluated these researches in general and concluded that the prophylactic use of antibiotics can lower the frequency of exacerbations in patients with COPD [10, 11]. However, several questions still remain unanswered, such as the most appropriate drug, the best regimen for its administration, and the most suitable target population. In the last two years, several well-designed and well-executed RCTs that investigated the effects of azithromycin have been newly published. Hence, in order to add more information and evidence to clinical practice, we performed an updated meta-analysis to evaluate the efficacy and safety of prophylactic macrolide therapy on the prevention of AECOPDs.

## Materials and Methods

### Search strategies

We systematically searched PubMed, Embase and the Cochrane Library from their inception until September 30th 2014 using the following search term: (COPD OR Chronic Obstructive Pulmonary Disease) AND (azithromycin OR erythromycin OR clarithromycin OR dirithromycin OR roxithromycin OR telithromycin OR macrolide). No language or time restrictions were applied. In addition, the reference lists of reports identified by this search strategy were also searched to select relevant articles.

### Selection Criteria

Studies were considered eligible for inclusion if they were RCTs that enrolled adults (older than 18 years of age) with a diagnosis of stable COPD. The diagnosis must have been confirmed with lung function testing (FEV_1_/FVC < 70%, FEV_1_ < 80% predicted, and an increase in FEV_1_ < 12% (or < 200 ml) after inhaling bronchodilators) [12]. The prophylactic use of macrolides must have been administered orally in appropriate daily doses of at least one time a week for a period of at least 3 months. Studies including patients with bronchiectasis, asthma, cystic fibrosis and other genetic diseases were excluded. Two investigators (Ni and Shao) independently performed the literature search and the study selection. Any disagreement was resolved by a third author (Cai), and a final consensus was reached among all authors.

### Data Extraction and Quality Assessment

The following variables were collected from the included studies by 2 independent reviewers: authors, publication year, country, year of the study, study design, number of patients (enrolled, intention-to-treat and clinically evaluable), main characteristics of the study population such as age, sex, smoking history, severity of COPD (the GOLD stage), type, dose and duration of macrolides administered, concomitant medication used to treat COPD, and information on outcome measures. Since controversy still exists as to the best method of reporting and analyzing data from clinical trials designed to reduce AECOPDs [6], we chose both an unweighted approach (total number of patients with one or more exacerbations) and a weighted approach (rate of exacerbations per patient per year) as primary outcomes. We accepted the definitions of exacerbation as reported in each study, since it was not possible to get data of all the patients that were included. The secondary outcomes were hospitalization rates, health-related quality of life using an accepted measure such as St George Respiratory Questionnaire (SGRQ) score, mortality, the total number of adverse events, and drug resistance. A Jadad score was used to assess the quality of RCTs included in this meta-analysis.

### Statistical Analysis

All statistical analyses were done with Comprehensive Meta-Analysis V2.2 (Biostat, Englewood NJ). We pooled the treatment results from the intention-to-treat data and calculated risk ratios (RR) with 95% CI for all clinical end points using random-effects (DerSimonian and Laird’s method) models. The between-study heterogeneity was assessed by the *I* ^*2*^ test (*I* ^*2*^ > 50% indicating the statistical inconsistency; >75% indicating heterogeneity). Sensitivity analyses were performed by deleting studies with the highest weight or a low Jadad score (<3). The causes of heterogeneity were explored by subgroup analyses such as type of macrolides and duration of therapy. Egger regression were used to evaluate publication bias, and *P* < 0.05 was considered statistically significant.

## Results

### Study characteristics

**Fig 1:**
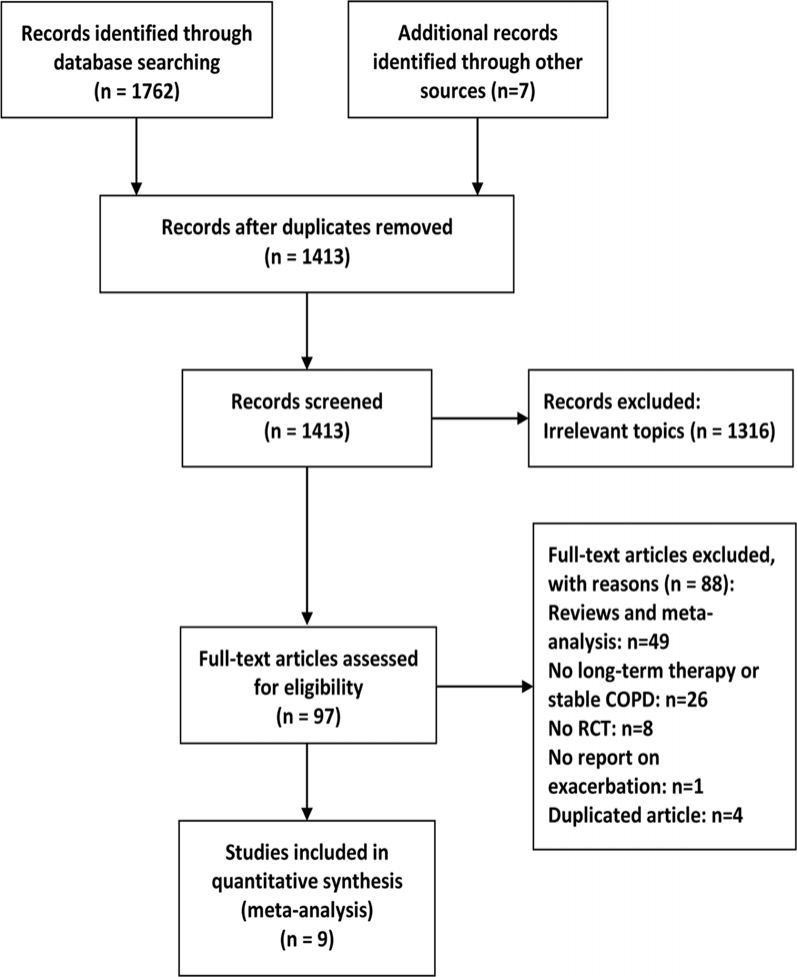
Flow diagram of included studies.

A total of 1762 potentially relevant reports were initially identified by PubMed, Embase, and the Cochrane Library searches (Fig. 1). Reference lists of reports were also searched to select relevant articles, yielding 7 further studies for evaluation. Briefly, the titles and abstracts were reviewed to exclude irrelevant studies. Ninety-seven articles with full texts were screened and 9 RCTs met the inclusion criteria. The characteristics of included studies are presented in Table 1. The 9 RCTs included 1666 patients, with ages generally ranging from 65–75 [13–21]. Most of them had moderate to severe COPD and had experienced exacerbations before study entry. A total of 830 patients were randomly allocated to the macrolide treatment group (1 study for clarithromycin, 3 for erythromycin and 5 for azithromycin) and 836 were randomly allocated to the control group. The study duration lasted for 3 months to 12 months and all used intention-to-treat analysis. One study did not mention the concomitant medication of recruited patients [16]. Two studies included patients requiring aerosolized antibiotics or systemic steroids [19, 21] and 1 study recruited patients who had been treated with theophylline and inhaled anticholinergic agents but not inhaled corticosteroids [13].

**Table 1:** Characteristics of studies included in the meta-analysis.

Author (Year)	Type of study	Number of patients (Treatment/Control)	Population Characteristics (Treatment/Control)			Type of macrolide	Antibiotic regimen	Duration of treatment	Concomitant medication to treat COPD	Jadad score
			Mean age	Pre-bronchodilator FEV1% predicted	Pre-bronchodilator FEV1/FVC % predicted					
Suzuki (2001) [13]	RCT	55/54	69.1/71.7	NA	NA	Erythromycin	200–400 mg once daily	12 months	Sustained release theophylline and inhaled anticholinergic agents, except corticosteroids	2
Banerjee (2005) [14]	RCT	31/36	65.1/68.1	42.5/43.9	NA	Clarithromycin	500 mg once daily	3 months	Inhaled corticosteroids	3
Seemungal (2008) [15]	RCT	53/56	66.5/67.8	49.3/50.6	48.9/50.9	Erythromycin	250 mg twice daily	12 months	Inhaled corticosteroids	5
Blasi (2010) [16]	RCT	11/11	72/73	NA	NA	Azithromycin	500 mg once 3 days/week	6 months	NA	2
He (2010) [17]	RCT	18/18	68.8/69.3	44.3/42.1	46.9/48.6	Erythromycin	125 mg 3 times daily	6 months	Inhaled corticosteroids, theophylline, inhaled anticholinergic agents, inhaled β-adrenergic agents	4
Albert (2011) [18]	RCT	558/559	65/66	39/40	42/43	Azithromycin	250 mg once daily	12 months	Inhaled corticosteroids, inhaled anticholinergic agents, inhaled β-adrenergic agents	3
Berkhof (2013) [19]	RCT	42/42	67/68	49.8/47.4	42.2/43.2	Azithromycin	250 mg once 3 days/week	3 months	Inhaled corticosteroids, inhaled anticholinergic agents, inhaled β-adrenergic agents, aerosolized antibiotics	5
Simpson (2014) [20]	RCT	15/15	71.7/69.9	56.5/51.1	52.3/51.3	Azithromycin	250 mg once daily	3 months	Inhaled corticosteroids	5
Uzun (2014) [21]	RCT	47/45	64.7/64.9	44.2/45.0	38.0/40.3	Azithromycin	500 mg once 3 days/week	12 months	Inhaled corticosteroids, inhaled anticholinergic agents, inhaled β-adrenergic agents, prednisolone	5

RCT, randomized controlled trial; NA, not applicable.

### Primary outcomes

**Fig 2:**
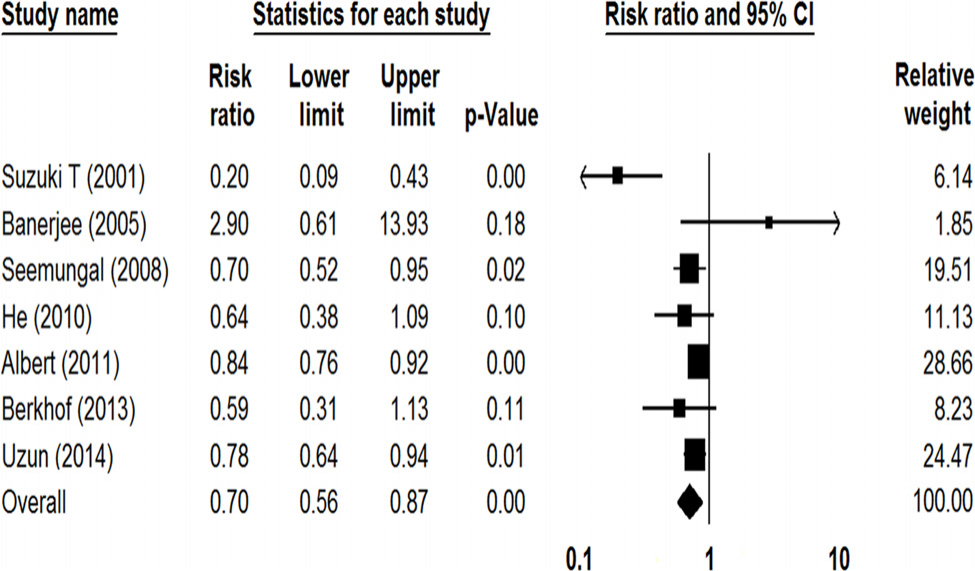
Forest plot of risk ratios for total number of patients with one or more exacerbations treated with macrolides compared with the control.

Seven studies involving 1614 participants reported the number of patients with one or more exacerbations [13–15, 17–19, 21]. As shown in Fig. 2, prophylactic macrolide treatment significantly reduced AECOPDs in comparison with the control group (RR = 0.70; 95% CI: 0.56–0.87; *P* < 0.01, *I* ^*2*^ = 66.43%). As the Jadad score of Suzuki et al. study was low [13], a sensitivity analysis was conducted with the other 6 RCTs, showing a 20% relative risk reduction of exacerbations among patients taking macrolides (RR = 0.80, 95% CI: 0.72–0.88, *P* < 0.01, *I* ^*2*^ = 12.47%).

**Fig 3:**
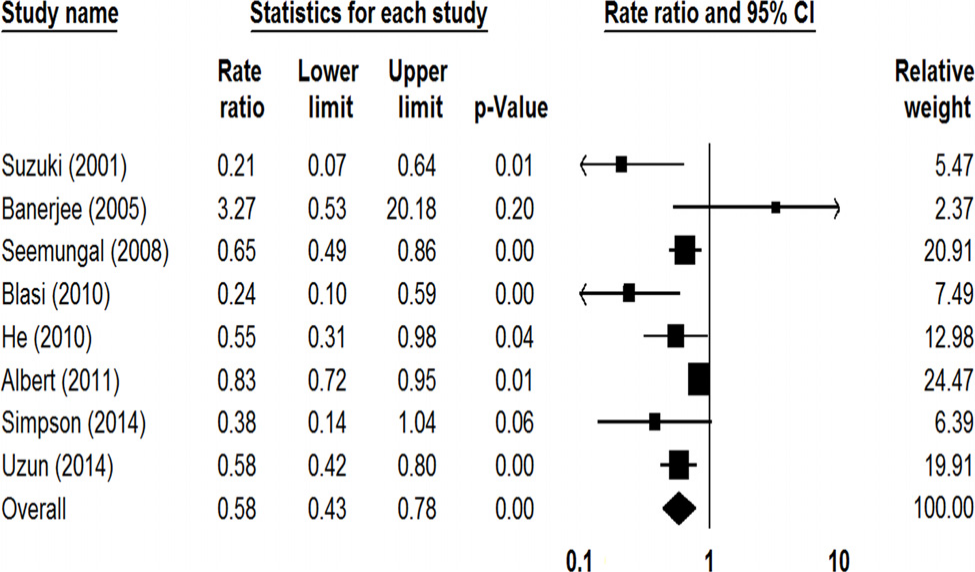
Forest plot of risk ratios for exacerbations per patient per year treated with macrolides compared with the control.

Eight studies involving 1582 participants reported the rate of exacerbations per patient per year [13–18, 20, 21], showing the prophylactic use of macrolides led to a reduction in the rate of exacerbations (RR = 0.58, 95% CI: 0.43–0.78, *P* < 0.01, *I* ^*2*^ = 67.80%; Fig. 3). The sensitivity analysis conducted by deleting the study with the highest weight (Albert et al.) found a 48% relative risk reduction of exacerbations among patients taking macrolides (RR = 0.52, 95% CI: 0.37–0.72, *P* < 0.01, *I* ^*2*^ = 48.39%) [13–17, 20, 21].

Subgroup analyses by the types of macrolide were shown in Table 2. Both clarithromycin and azithromycin therapy for 3 months showed no statistical differences between the treated and control groups, whereas both clarithromycin and azithromycin therapy for 6–12 months significantly reduce the number of exacerbations or the rate of exacerbations per patient per year. In addition, 1 study which had adopted a daily dosing regimen of azithromycin therapy for 12 months, found a reduction in the rate of exacerbations (RR = 0.83, 95% CI: 0.72–0.95, *P* = 0.01) [18]. Two studies which had adopted an intermittent regimen of azithromycin therapy for 6–12 months, also found a reduction in the rate of exacerbations (RR = 0.41, 95% CI: 0.18–0.96, *P* = 0.04, *I* ^*2*^ = 69.32%) [16, 21].

**Table 2:** Subgroup analyses of prophylactic macrolide treatment on the prevention of acute exacerbations of COPD.

Variables (macrolide)	Number of patients with exacerbations			Rate of exacerbations per patient per year		
	Studies (patients), No.	RR (95% CI)	*P*	Studies (patients), No.	RR (95% CI)	*P*
Clarithromycin 3 months	1 (67)	2.90 (0.61–13.93)	0.18	1 (67)	3.27 (0.53–20.18)	0.20
Azithromycin 3 months	1 (84)	0.46 (0.18–1.18)	0.11	1 (30)	0.38 (0.14–1.05)	0.06
Azithromycin 6–12 months	2 (1209)	0.82 (0.76–0.90)	0.00	3 (1231)	0.59 (0.37–0.93)	0.02
Erythromycin 6–12 months	3 (254)	0.49 (0.26–0.91)	0.02	3 (254)	0.53 (0.43–0.83)	0.01

RR, risk ratio

### Secondary outcomes

**Fig 4:**
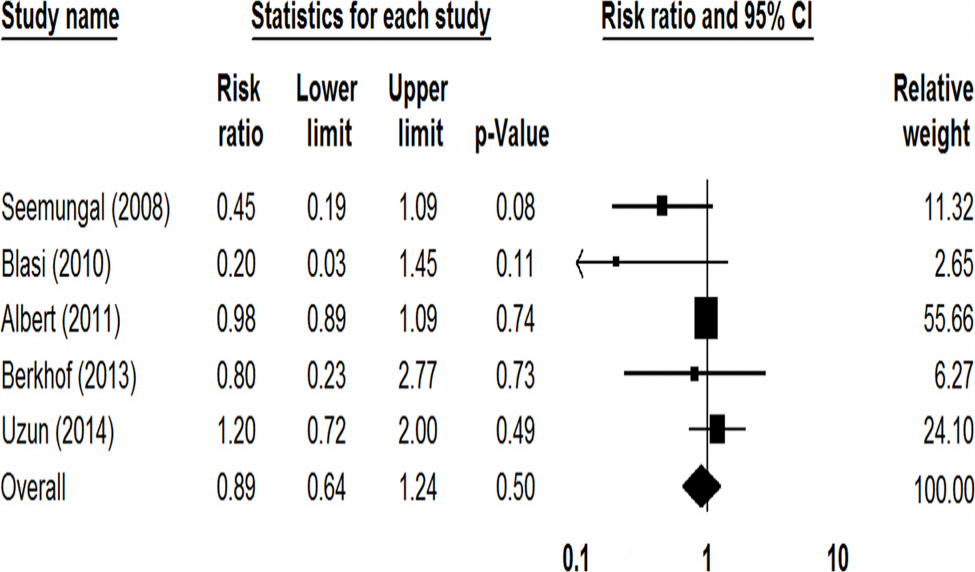
Forest plot assessing risk ratio of hospitalization among COPD patients treated with macrolides compared with the control.

The overall number of hospitalizations due to AECOPDs was reported in 5 studies involving 1424 patients [15, 16, 18, 19, 21], and the rate of death was reported in 3 studies involving 1248 patients [13, 16, 18]. No significant difference was observed between the macrolide therapy and control groups in the overall number of hospitalizations (RR = 0.89, 95%CI: 0.64–1.24, *P* = 0.50, *I* ^*2*^ = 34.65%; Fig. 4) or in the all-cause rate of death (RR = 0.66, 95%CI: 0.23–1.85, *P* = 0.43, *I* ^*2*^ = 22.95%).

**Fig 5:**
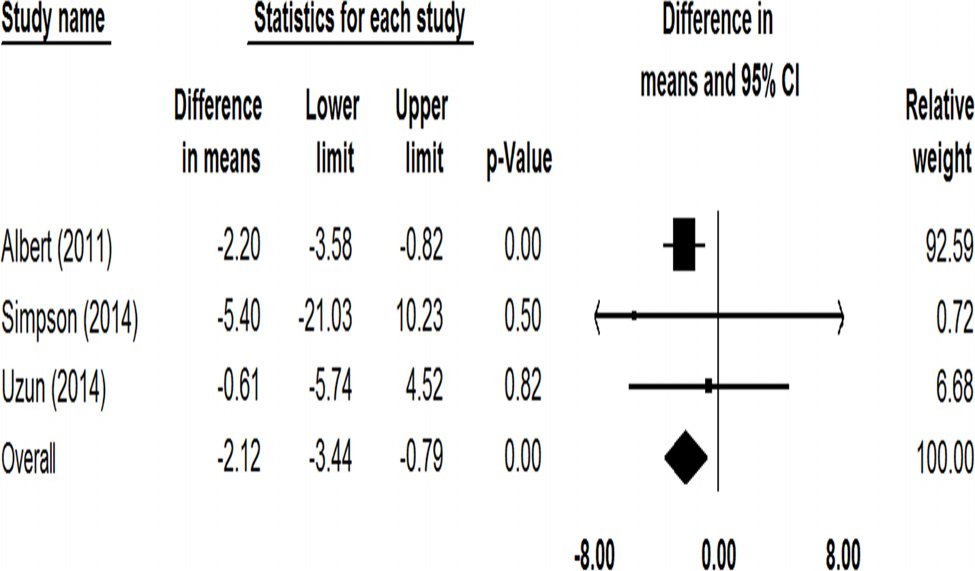
Forest plot of comparing the mean differences (MD) in change of SGRQ total score among COPD patients treated with macrolides compared with the control.

In terms of health-related quality of life, 4 studies including 1323 patients reported changes of SGRQ total score before and after azithromycin treatment [18–21]. There was very high heterogeneity among these studies (*I* ^*2*^ = 97.10%). The change of SGRQ total score in the study of Berkhof et al. [19] was much greater than in the other 3 studies, thus, a sensitivity analysis was performed. The prophylactic use of azithromycin could significantly reduce the SGRQ total score (Mean difference = -2.12, 95% CI: -3.44 to -0.79, *P* = 0.002, *I* ^*2*^ = 0%; Fig. 5).

**Fig 6:**
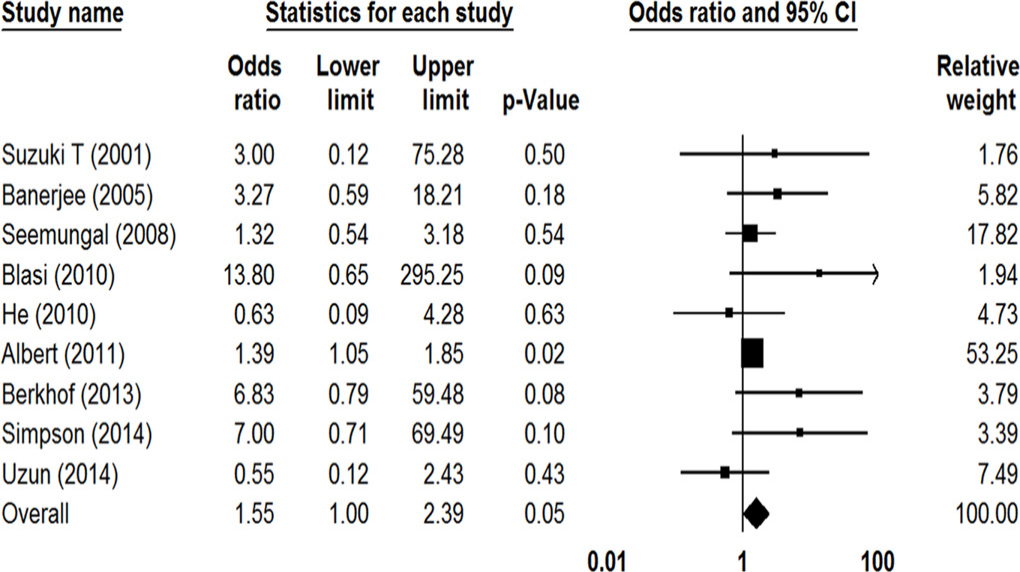
Forest plot of odds ratios for drug adverse effects in COPD patients treated with macrolides compared with the control.

All included studies reported the total number of adverse events that had occurred in both the prophylactic macrolide treatment group and control group. As shown in Fig. 6, there was a tendency for more adverse events in the treatment group (OR = 1.55, 95%CI: 1.003–2.39, *P* = 0.049, *I* ^*2*^ = 15.04%). In subgroup analysis, 3 studies that had used erythromycin [13, 15, 17] showed no statistical difference between two groups (OR = 1.22, 95%CI: 0.56–2.66, *P* = 0.61, *I* ^*2*^ = 0%), and nor did 5 studies using azithromycin (OR = 2.08, 95%CI: 0.80–5.37, *P* = 0.13, *I* ^*2*^ = 47.21%) [16, 18–21]. Gastrointestinal reactions were the most frequent adverse events in the treatment groups. Albert et al. reported a rate of hearing reduction of 25% in the azithromycin group versus 20% in the placebo group (OR = 1.39; 95% CI: 1.05–1.85, *P* = 0.04) [18], However, the other 4 studies reported no occurrence of ototoxicity [16, 19–21]. Three studies including 212 patients reported 4 cardiovascular events in the treatment group and 2 in the placebo group (*P* = 0.43) [17, 19, 21]. One study found the rate of death due to cardiovascular events was 0.2% in both groups (*P* = 1.00) [18].

In addition, 6 studies involving 1491 patients monitored the susceptibility changes of macrolide during the treatment [14–16, 18, 19, 21]. Three studies respectively reported 1 case of macrolide resistance in the treatment group [15, 16, 19]. In the study of Albert et al. [18], susceptibility surveillance of pathogens isolated from patients newly colonized during the study period found the resistance rate was higher in the treatment group (*P* < 0.001). However, a recently published study of Uzun et al. reported different results. Macrolide-resistant bacteria was observed in 3 (6%) patients in the treatment group compared with 11 (24%) patients in the placebo group (*P* = 0.036) [21].

### Risk of bias in included studies

Six included studies reported on the methods of randomization and blinding [13–15, 19–21]. All but 2 studies were double blinded [13, 16]. There were no significant difference in the overall withdrawal rate between the macrolide therapy and control groups (7.71% vs. 6.46%, *P* = 0.32). Egger regression showed no significant publication bias among the included trials regarding overall results of all the primary and secondary outcomes.

## Discussion

Macrolides are a class of antibiotics characterized by the presence of a macrocyclic lactone ring [22]. They have great bioavailability, extensive tissue penetration and broad-spectrum antibacterial activity, making them effective for treating infectious respiratory diseases [23, 24]. In recent years, studies have found macrolides possess a variety of immune-modulatory and physiological properties as well, such as anti-inflammatory and anti-viral effects, reducing mucus secretion, and inhibiting bacterial virulence and biofilm formation [25]. Previous studies have shown that long-term macrolide therapy is effective in controlling diffuse panbronchiolitis and cystic fibrosis [26, 27]. Taking into consideration that the occurrence of AECOPDs is also associated with the aggravation of airway inflammation and infection, prophylactic macrolide therapy could be beneficial to patients with COPD [25].

By both unweighted and weighted approaches, the pooled evidence of our meta-analysis confirmed that the prophylactic macrolide therapy could significantly reduce the frequency of exacerbations in patients with COPD. In subgroup analysis, previous meta-analyses found only long-term erythromycin treatment lowered the frequency of exacerbations. Our study, which included more RCTs, indicated azithromycin therapy for 6–12 months was also effective. Regarding the health-related quality of life, pooled data of 2 trials on moxifloxacin and azithromycin showed statistically significant reduction in SGRQ total score, with no heterogeneity [28]. However, in our study, we observed very high heterogeneity among 4 trials involving azithromycin. The most likely reason is that the study of Berkhof et al. specially recruited patients suffering from chronic productive cough [19]. Therapy with azithromycin improved cough-specific health status and thus significantly reduced the SGRQ total score [19]. Sensitivity analysis of the other 3 studies also found a statistically significant reduction of SGRQ in the treatment group, but not achieving the level of clinical significance (≥4-point reduction).

So far, the optimal regimen of macrolide treatment has not been well established. Different durations and dosages were adopted among the studies included in this meta-analysis. For azithromycin, 3 studies using 1500–1750 mg/week for 6–12 months observed a significantly lower frequency of exacerbations in the treatment groups [16, 18, 21], while the other 2 studies using 750 or 1750 mg/week for 3 months found no reduction of exacerbations [19, 20]. This indicated that a relative shorter-term therapy might not bring significant benefits. In addition, the question of whether intermittent prophylaxis is as effective as daily dosing in preventing exacerbations is unknown. In controlling cystic fibrosis, an intermittent regimen is the preferred starting regimen, with a transition to daily dosing if intermittent dosing appears ineffective [29]. Our meta-analysis showed that both the intermittent and daily dosing regimen of azithromycin therapy for 6–12 months could significantly reduce the rate of COPD exacerbations. Moreover, the most appropriate time to commence prophylactic therapy deserves further investigation. Gómez J et al. found patients with COPD who received azithromycin for 3 days every 21 days during the winter periods experienced much less exacerbations [30]. Considering that acute exacerbations of COPD more frequently occur during periods with low temperature such as the cold winter and spring [31], the timing of prophylactic therapy probably should cover these periods.

Though a series of studies have confirmed the immune-modulatory and physiological properties of macrolides, the concrete mechanisms of action are incompletely understood [32]. In vitro studies showed these properties were only observed among macrolides with 14 and 15-membered macrocyclic lactone ring such as erythromycin, clarithromycin, roxithromycin and azithromycin [33]. The effects of clarithromycin and roxithromycin on the inhibition of inflammatory cytokine production by COPD sputum cells was more potent than that of azithromycin [34]. In this meta-analysis, the results of studies that included erythromycin and azithromycin were high consistency, but 1 study on clarithromycin found 3 months treatment could not effectively prevent AECOPDs. Although the power of this study was limited by a short study period and the small number of total exacerbations, it would be worth further investigation to determine whether all types of 14 and 15-membered macrolides possess the same efficacy in preventing AECOPDs.

Another crucial question that still remains unanswered would be to define the target population of COPD patients who can benefit from the use of long-term antibiotics. As a result of different inclusion criteria and limited reported data in these RCTs that were included, we could not determine which patients with COPD would be the most suitable for receiving the prophylactic use of a macrolide. In the large pivotal study of azithromycin, Han and colleagues reported that azithromycin had greater efficacy in older patients and milder GOLD stages, with little evidence of treatment effect among current smokers after adjusting for potentially relevant confounders [35]. The recent Spanish COPD guidelines suggest that long-term treatment with macrolides should be considered in patients with severe COPD who despite optimal pharmacologic and non-pharmacologic treatment, have frequent exacerbations or hospital admissions [36]. In order to determine the most appropriate target population and prevent the excessive use of long-term antibiotics in the community, further studies are still required.

It has been suggested that long-term macrolide therapies have several side effects, especially gastrointestinal reactions [25]. Our meta-analysis showed a tendency toward more adverse events in the treatment group. Compared with other 4 studies using azithromycin, Albert et al. observed higher hearing impairment in both the treatment and placebo groups [18]. Although previous studies have indicated that the use of macrolides in the setting of AECOPDs may lead to increased cardiovascular events [37–39], the occurrence of cardiovascular events was lower in the studies we included. This might be due to the likelihood that most studies specially recruited patients without cardiovascular disorders. Another major concern surrounding the long-term use of macrolides is the emergence of antibiotic resistance. Though only a small number of patients developed resistance during macrolide treatment, the susceptibilities of respiratory pathogens to macrolides should be closely monitored.

It should be noted that several limitations exist in our meta-analysis. The main limitations are that the studies we included had different inclusion criteria, used differing concomitant medications, and employed varied durations and dosages of macrolides. As a result, there is a degree of clinical heterogeneity between these studies. Another limitation is that we could not perform further analysis on the occurrence of specific adverse events because of the lack of standard criteria for assessing adverse events. In addition, the sample size for certain subgroup analyses was small, which has reduced the power of statistical analysis.

In summary, our meta-analysis confirmed long-term erythromycin or azithromycin therapy could significantly reduce the frequency of exacerbations in patients with COPD. However, long-term usage may bring increased adverse events and the emergence of macrolide-resistance. Recommendations of prophylactic macrolide therapy should be determined on a case-by-case basis by fully weighing the clinical benefits and the potential risks.

## Supporting Information

S1 Checklist PRISMA 2009 checklist. (DOC)
